# Mechanisms through which table tennis practice influences the development of big five personality traits in primary school students

**DOI:** 10.3389/fpsyg.2026.1705050

**Published:** 2026-01-28

**Authors:** Jian Wang, Yating Zou, Liping Du, Yang Chen, Guoyuan Huang

**Affiliations:** 1 School of Physical Education and Health, East China Jiaotong University, Nanchang, China; 2 College of Education, Nanchang Vocational University, Nanchang, China; 3 School of Physical Education, Xichang University, Xichang, China; 4 School of Physical Education, Xi'an Jiaotong University, Xi'an, China; 5 College of Physical Education, Sichuan Agricultural University, Ya'an, China

**Keywords:** big five personality traits, exercise intervention, primary school students, psychology, table tennis

## Abstract

This quasi-experimental study examined the effects of a 16-week systematic table tennis training program on the Big Five personality traits of primary school students. Previous research has largely focused on the macro-level effects of physical activity, with limited fine-grained investigation into the psychological shaping mechanisms of specific sports, particularly intervention-based evidence involving children without prior training. A total of 98 male students aged 9–10 years were enrolled and, after propensity score matching (including age, baseline personality scores, etc.), were allocated to an experimental group (*n* = 49) or a control group (*n* = 49). The experimental group received moderate-intensity table tennis training three times per week (120 min per session), comprising technical drills, tactical exercises, and match simulations, while the control group maintained their routine activities. The short-form NEO Five-Factor Inventory (NEO-FFI) was administered before and after the intervention. A 2 × 2 mixed-design ANOVA and analysis of covariance (ANCOVA), with pretest scores as covariates, were used to analyze the effects. The results revealed significant Time × Group interaction effects for Openness (*F*(1,92) = 64.32, *p* < 0.001, *η*^2^ = 0.411), Neuroticism (*F*(1,92) = 42.18, *p* < 0.001, *η*^2^ = 0.314), Agreeableness (*F*(1,92) = 89.45, *p* < 0.001, *η*^2^ = 0.493), and Extraversion (*F*(1,92) = 56.74, *p* < 0.001, *η*^2^ = 0.381) but not for Conscientiousness (*p* = 0.076). Simple effects analyses indicated that the experimental group showed significant post-intervention improvements in Openness (*d* = 1.67), Agreeableness (*d* = 1.56), and Extraversion (*d* = 0.86), along with a significant reduction in Neuroticism (*d* = −0.89). No significant changes were observed in the control group. The findings suggest that systematic table tennis training can effectively promote the development of emotional stability, openness, agreeableness, and extraversion in primary school students, but it has a limited effect on conscientiousness. This study provides preliminary causal evidence for fostering children’s personality development through a specific sport and offers empirical support for the integration of sports and education. Limitations include the all-male sample and the quasi-experimental design. Future research should employ large-scale randomized controlled trials and multi-source assessments to further validate these results.

## 1 Introduction

Childhood and adolescence represent a critical period for personality formation and psychological health development. However, children in contemporary society commonly face multiple challenges such as increased academic pressure, excessive screen time, and insufficient structured physical activity. These factors may pose significant risks to their emotional regulation, social adaptability, and healthy personality development (Twenge and Campbell, 2018; Kandola et al., 2020). According to the World Health Organization and multiple transnational studies, the global prevalence of emotional and behavioral problems among children and adolescents is rising, highlighting an urgent need for effective, feasible, and engaging early interventions (World Health Organization, 2025; Racine et al., 2021). Regular physical activity is widely recognized as an effective means to promote psychosocial health in children (Biddle et al., 2019). Nevertheless, its benefits may vary depending on the intrinsic characteristics of the sport. Table tennis is an open-skill sport that demands rapid perceptual decision-making, fine motor control, and tactical planning, while also incorporating instantaneous social interactions (e.g., doubles coordination and opponent communication). This dynamic integration of cognitive load, emotional management, and social context may allow table tennis to extend beyond general physical exercise, providing a unique setting for cultivating emotional stability, cooperation and trust, social initiative, and cognitive flexibility (González-Devesa et al., 2024; Marta et al., 2025). Compared to sports with high repetitiveness or structurally simple social interactions, the continuous rally-based competition and strategic adjustments in table tennis resemble the concept of “tactical creativity,” offering participants intensive “micro-practice” opportunities to handle pressure, navigate social dynamics, and engage in creative thinking (Farrow and Abernethy, 2015). Therefore, investigating the effects of table tennis on children’s personality traits constitutes an important starting point for exploring the psychosocial benefits of specific sports.

The Five-Factor Model of personality (Openness, Conscientiousness, Extraversion, Agreeableness, and Neuroticism) provides a robust theoretical and measurement framework for quantifying such effects (McCrae and Costa, 2003). Research indicates a bidirectional relationship between personality traits and sports participation: Personality influences sport preference and adherence, while sustained sport experience may, in turn, shape the expression and development of personality traits (Wilson and Dishman, 2015; Stephan et al., 2018). For example, table tennis players who exhibit traits such as independence, emotional balance, quick reaction, extraversion, alertness, and communication skills demonstrate greater efficiency and stability in competition (Ahsan and Mohammad, 2017). In contrast, elite athletes in individual sports tend to show higher levels of conscientiousness (Steca et al., 2018). Given the high plasticity of personality during childhood and adolescence, longitudinal studies have shown systematic changes in traits such as conscientiousness and emotional stability from childhood to early adulthood. Parenting styles, family environment, and other factors can profoundly influence developmental trajectories, and the neurobehavioral system is particularly sensitive to experiences of regular structured activities. This offers a critical window for actively guiding personality development through systematic sports interventions (Casey et al., 2019; Roberts et al., 2006; De Haan et al., 2012).

Although the association between sports and personality has been established, existing research has notable limitations. First, most evidence comes from cross-sectional designs or comparisons between athletes and non-athletes, which limits causal inference (Allen et al., 2013). Second, studies often focus on the macro-level effects of “physical activity” and lack detailed investigation into the unique psychological shaping mechanisms of different sports (Vella et al., 2020; Egloff and Gruhn, 1996). Specifically in table tennis, while some studies have outlined the personality profiles of athletes—indicating that conscientiousness is the most prominent trait among table tennis players, characterizing them as individuals who pursue precision, organization, and perseverance to achieve goals (Marchese et al., 2022; Lopez and Santelices, 2012)—there remains a scarcity of intervention-based experimental research that systematically examines the causal impact of table tennis training on each dimension of the Five-Factor Model in children without prior training. This gap limits our ability to provide evidence-based recommendations for designing school physical education curricula and strategies aimed at promoting children’s mental health.

Building on this, the present study implemented a 16-week quasi-experimental design to examine the effects of systematic table tennis training on the Five-Factor Model personality traits of primary school students. It was hypothesized that, compared to the control group, children in the table tennis training group would demonstrate greater improvements in Extraversion, Agreeableness, and Openness, exhibit a greater reduction in Neuroticism, and show potential changes in the Conscientiousness dimension. The findings are expected to provide empirical evidence and theoretical insights for implementing targeted personality and mental health interventions during children’s critical developmental periods using table tennis, a sport with broad appeal.

## 2 Methods

### 2.1 Experimental design

This study employed a quasi-experimental, non-equivalent group design to explore the effects of table tennis practice on personality development in primary school students by comparing changes in Five-Factor Model personality traits between an experimental group and a control group before and after the intervention. To enhance intergroup comparability, propensity score matching was used to match participants across the two groups based on variables including age, gender, family socioeconomic status, baseline personality trait scores, and willingness to engage in physical activity. This approach aimed to minimize selection bias and control for potential confounding factors as effectively as possible.

### 2.2 Participants

A total of 98 participants were recruited from the Ya’an Starlight Club and the Chengdu Experimental Foreign Language Elementary School for this study. The sample primarily consisted of third- and fourth-grade students, aged approximately 9–10 years. The cohort included 49 trainees in the experimental group and 49 third-grade students in the control group. All participants were male. Participation was entirely voluntary, and all participants retained the right to withdraw from the study at any time, without condition or obligation. Participants could withdraw from the study due to physical or psychological discomfort, scheduling conflicts, or any other personal reason, without facing any questioning or impact on their school-related rights and interests. Necessary support was to be provided immediately in case any discomfort arose during the study. Data from participants who withdrew were removed in accordance with regulations, fully respecting their rights regarding data handling. The study adhered to the principles of the Declaration of Helsinki. Emergency equipment and medical support were available during training sessions, and all coaches had received basic first-aid training. No serious sports injuries occurred during the study; minor discomforts (for example, muscle soreness) were promptly addressed and documented. Informed consent was obtained from both the participants and their parents or guardians. The study protocol was reviewed and approved by the Ethics Committee of Sichuan Agricultural University (Approval No. H20250039).

The participants were divided into an experimental group and a control group, matched for age. Those in the experimental group were randomly selected from newly enrolled club members with no prior training experience (i.e., beginners with no systematic exposure to the sport). The participants in the control group were randomly selected from regular, naturally formed classes. The control group maintained their routine daily activities and did not receive any organized sports intervention as part of the experiment. To control for potential “extra-attention” effects, the participants in the control group were permitted to engage in other low-intensity or non-competitive physical activities. During the study period, the experimental group underwent systematic table tennis training three times per week, with each session lasting 2 h. The training session structure consisted of four parts: Warm-up (15 min), technical drills (40 min), tactical training and match simulation (45 min), and cool-down with a summary (20 min). Polar heart rate monitors were used to record average heart rate, with a target zone of 120–150 bpm to ensure moderate exercise intensity. An attendance rate of ≥85% was required for valid participation. The average attendance rate for the experimental group in this study was 92.3%. All coaches held national table tennis coaching certifications and received unified training to ensure consistency in instruction. Furthermore, every 2 weeks, the experimental group participated in competitions, such as youth league matches on weekends.

### 2.3 Experimental methods

#### 2.3.1 Experiment time and location

(1) Duration: 1 March 2025–25 June 2025.
(2) Location: Chengdu Experimental Foreign Language Elementary School.

#### 2.3.2 Experimental scale

The NEO Five-Factor Inventory (NEO-FFI) was used to assess personality traits. Since its revision, this scale has been widely used to measure the Big Five personality traits among Chinese adolescents (Nie et al., 2008). The questionnaire consists of five dimensions: Neuroticism, Extraversion, Openness, Agreeableness, and Conscientiousness. Each dimension includes 12 items, resulting in a total of 60 items. Responses are recorded on a 5-point Likert scale (1 = strongly disagree, 2 = disagree, 3 = uncertain, 4 = agree, and 5 = strongly agree). In the current study, Cronbach’s *α* coefficients for each dimension were as follows: Extraversion 0.81, Agreeableness 0.79, Conscientiousness 0.83, Neuroticism 0.77, and Openness 0.80. The overall scale demonstrated good internal consistency, with a total *α* of 0.88.

#### 2.3.3 Experimental hypotheses

Main Hypothesis: Systematic table tennis training can positively influence the Big Five personality traits of primary school students. The experimental group is expected to show significantly better scores on personality traits post-intervention compared to the control group.

Sub-hypotheses: The experimental group is expected to score significantly higher than the control group on the Conscientiousness dimension (including subdimensions such as Orderliness, Responsibility, and Self-discipline). Conversely, the experimental group is expected to score significantly lower than the control group on the Neuroticism dimension (including subdimensions such as Anxiety, Hostility, and Emotional Volatility), indicating greater emotional stability. In addition, the experimental group is expected to score significantly higher than the control group on the Openness dimension (including subdimensions such as Curiosity, Imagination, and Willingness to try new things). The experimental group is also expected to score significantly higher than the control group on the Agreeableness dimension (including subdimensions such as Trust in others, Cooperativeness, and Altruistic behavior). Finally, the experimental group is expected to score significantly higher than the control group on the Extraversion dimension (including subdimensions such as Gregariousness, Activity level, and Positive emotions).

#### 2.3.4 Experimental variables

(1) Independent Variable: The experimental group received professional table tennis training.
(2) Dependent Variables: Scores on the personality trait dimensions for both the experimental and control groups.
(3) Control variables:
(1) Group Matching Control: As shown in Table 1, SPSS 23.0 was used for the statistical analysis of pretest data. Independent samples *t*-tests indicated no significant differences between the two groups (*p* > 0.05), confirming that all Big Five personality dimensions were at comparable levels prior to the intervention.
(2) Instructor and Coach Control: The author monitored all classroom teaching and table tennis training sessions to minimize interference from extraneous sports-related factors and reduce potential impacts on experimental outcomes.
(3) Posttest Validation: Pre- and post-intervention comparisons of the control group’s Big Five personality scores were conducted to assess whether any changes occurred. Any observed differences would indicate that control variables or the entire experiment were influenced by external factors.

**Table 1:** Baseline comparison across the five dimensions before intervention.

Dimension	Groups	*N*	*M* ± SD	*T*	*P*
Neuroticism	Experimental group	47	13.31 ± 0.35	−1.12	0.265
	Control group	49	13.38 ± 0.24		
Conscientiousness	Experimental group	47	24.28 ± 0.86	1.34	0.184
	Control group	49	24.04 ± 0.84		
Agreeableness	Experimental group	47	11.18 ± 0.42	0.45	0.665
	Control group	49	11.14 ± 0.45		
Openness	Experimental group	47	36.37 ± 0.89	0.89	0.376
	Control group	49	36.21 ± 0.81		
Extraversion	Experimental group	47	20.87 ± 0.68	1.34	0.184
	Control group	49	20.30 ± 0.78		

### 2.4 Data analysis

Due to two participants in the experimental group withdrawing from the study because of illness, the final sample included 96 participants (*n* = 96), with 47 in the experimental group (*n* = 47) and 49 in the control group (*n* = 49). Data analysis was performed using the SPSS 23.0 statistical software. Descriptive statistics (*M* ± SD) were calculated for each variable, and baseline equivalence tests were conducted. Normality was assessed using the Shapiro–Wilk test, and homogeneity of variance was examined using Levene’s test. A 2 × 2 mixed-design ANOVA (Group × Time) was employed to test interaction effects and simple effects. Analysis of covariance (ANCOVA) was also conducted using pretest scores as covariates. Effect sizes were reported accordingly, with η^2^ for ANOVA and Cohen’s d for between-group comparisons.

## 3 Results

### 3.1 Inferential statistical analysis of intervention effects

Prior to conducting inferential analyses, Shapiro–Wilk tests for normality and Levene’s tests for homogeneity of variance were performed on the pretest data for each dimension (see Table 2). The normality test results indicated that the pretest data for all dimensions followed a normal distribution (all *p* > 0.05). The Levene’s test results confirmed that the assumption of homogeneity of variance was met for all dimensions (all *p* > 0.05). In summary, the data satisfied the prerequisites for parametric testing, allowing for the subsequent use of analysis of variance.

**Table 2:** Results of the Shapiro–Wilk test and Levene’s test.

Dimension	Groups	*N*	*W*	*P*	*F*	*P*
Neuroticism	Experimental group	47	0.97	0.087	0.12	0.730
	Control group	49	0.98	0.415		
Conscientiousness	Experimental group	47	0.98	0.314	0.67	0.415
	Control group	49	0.97	0.103		
Agreeableness	Experimental group	47	0.98	0.253	0.89	0.348
	Control group	49	0.96	0.052		
Openness	Experimental group	47	0.98	0.326	0.45	0.503
	Control group	49	0.97	0.125		
Extraversion	Experimental group	47	0.97	0.066	0.03	0.865
	Control group	49	0.97	0.089		

### 3.2 Results of mixed-design analysis of variance

To examine the intervention effects of table tennis training on the Big Five personality traits of the primary school students, a 2 (Group: experimental/control) × 2 (Time: pretest/posttest) mixed-design analysis of variance (ANOVA) was conducted, with a primary focus on the “Time × Group” interaction effect.

**Figure 1:**
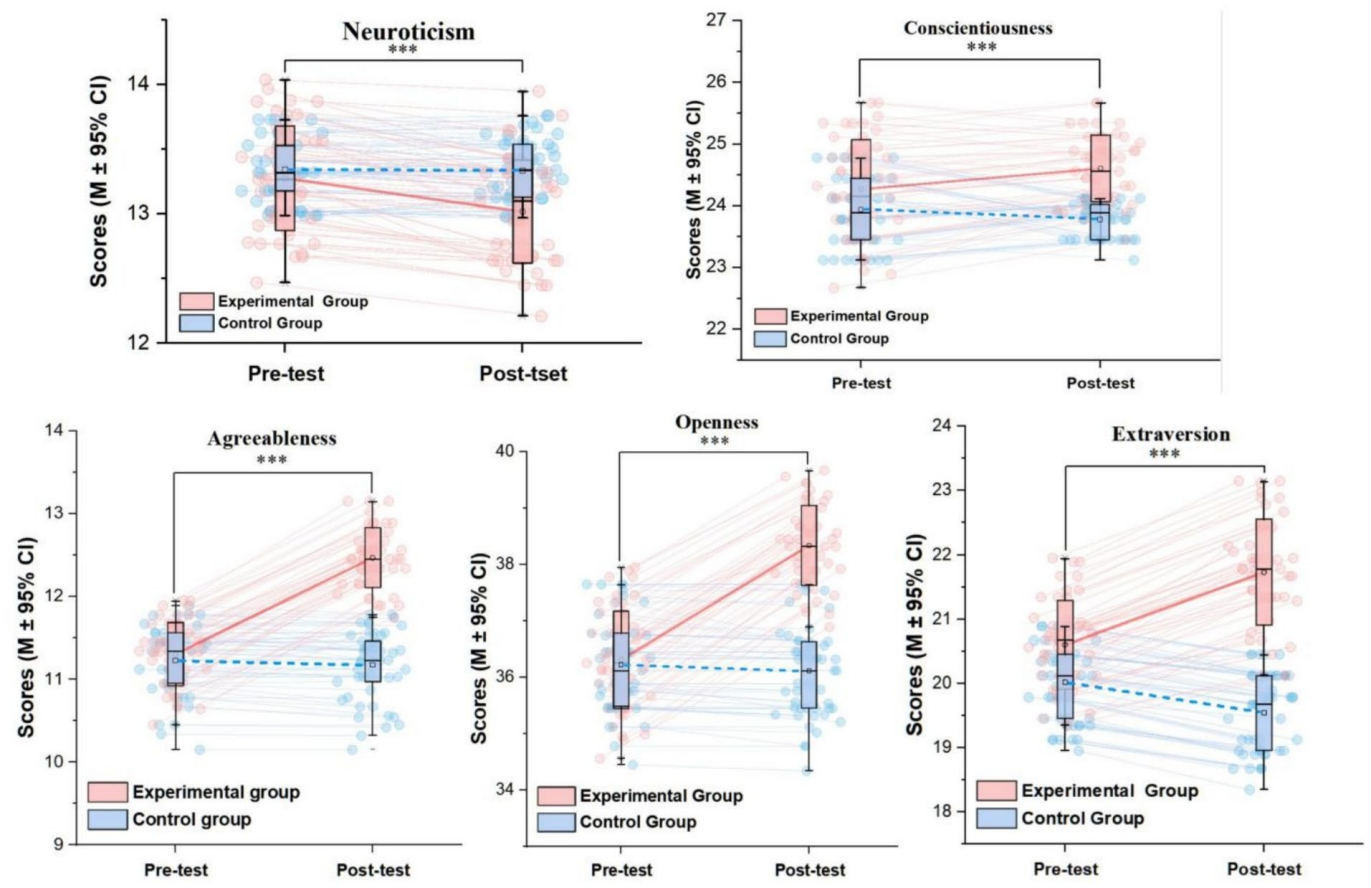
Time × Group interaction effects across the five dimensions of the big five personality traits.

The results of the mixed-design ANOVA (details in Table 3 and Figure 1) revealed significant “Time × Group” interaction effects for the following four dimensions: Openness, Neuroticism, Agreeableness, and Extraversion (*all p* < 0.001). This indicates that table tennis training exerted specific effects on the changing trends of these personality traits. Among these, the interaction effect was largest for the Agreeableness dimension (partial *η*^2^ = 0.493). In contrast, the interaction effect for the Conscientiousness dimension did not reach statistical significance (*p* = 0.076), suggesting that the training did not produce a distinct pattern of change in this trait compared to the control group.

**Table 3:** Results of mixed-design ANOVA (time × group interaction effect).

Dimension	*F*	Degrees of freedom (df)	*P*	Partial *η*^2^
Neuroticism	42.18	1, 92	<0.001	0.314
Conscientiousness	3.21	1, 92	0.076	0.034
Agreeableness	89.45	1, 92	<0.001	0.493
Openness	64.32	1, 92	<0.001	0.411
Extraversion	56.74	1, 92	<0.001	0.381

### 3.3 Results of analysis of covariance (ANCOVA)

To further control for potential influences of pretest baseline scores and enhance the precision of statistical testing, analysis of covariance (ANCOVA) was conducted, with the posttest scores for each dimension as the dependent variable and the corresponding pretest scores as the covariate.

The results (see Table 4) confirmed that, after controlling for pretest scores, the experimental group still scored significantly higher than the control group on Openness, Agreeableness, and Extraversion at posttest, while scoring significantly lower on Neuroticism (all *p* < 0.001). Effect size analysis showed that the between-group difference was most pronounced for Agreeableness (partial *η*^2^ = 0.404). Consistent with previous findings, the Conscientiousness dimension again showed no significant group effect (*p* = 0.092), further supporting the conclusion that table tennis training had a limited impact on this trait.

**Table 4:** Results of analysis of covariance (Main effects by group).

Dimension	*F*	Degrees of freedom (df)	*P*	Partial *η*^2^
Neuroticism	25.34	1, 91	<0.001	0.216
Conscientiousness	2.89	1, 91	0.092	0.030
Agreeableness	62.45	1, 91	<0.001	0.404
Openness	38.76	1, 91	<0.001	0.296
Extraversion	41.23	1, 91	<0.001	0.310

### 3.4 Simple effect analysis and effect size calculation results

**Figure 2:**
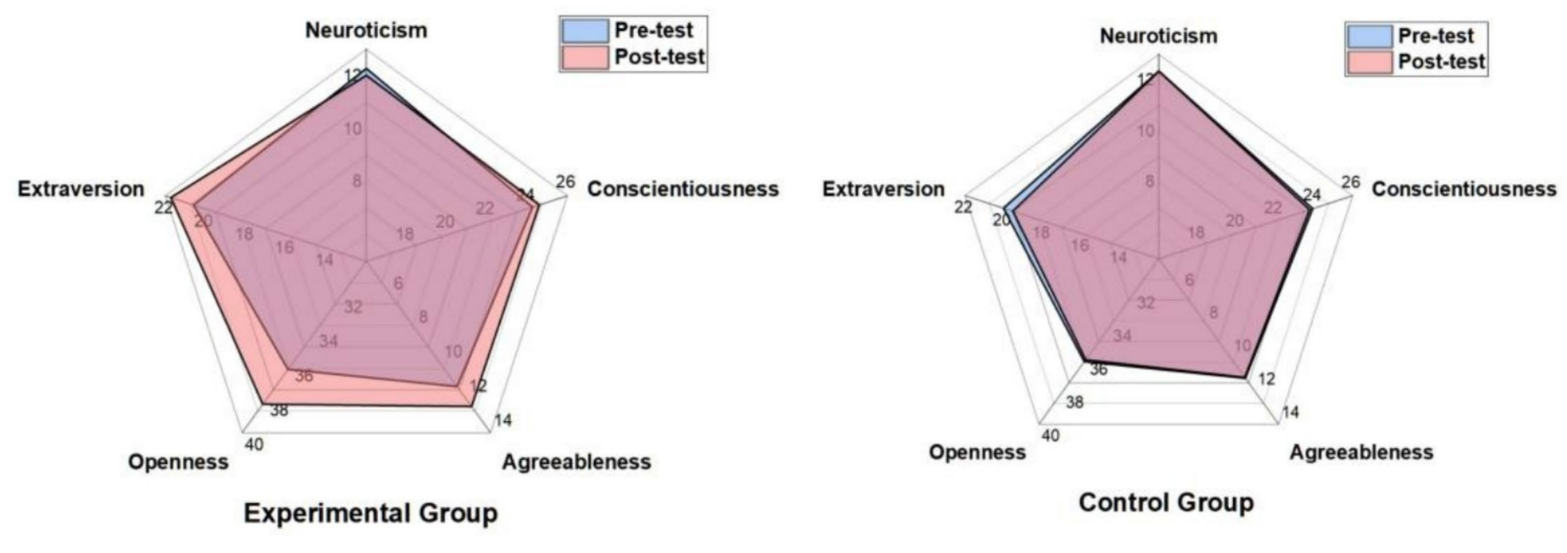
Changes in big five personality trait scores before and after the experiment in the control and experimental groups.

Simple effects analysis (see Table 5) clarified the source of the interaction effects. Following the intervention, the experimental group showed significant increases in Openness, Agreeableness, and Extraversion compared to their pretest scores, with large effect sizes (Cohen’s d ranging from 0.86 to 1.67). Concurrently, the experimental group’s Neuroticism score decreased significantly (*d* = −0.89), indicating enhanced emotional stability. In contrast, the control group showed no significant positive changes from pretest to posttest on these dimensions; notably, a slight decline was observed for Extraversion. These results clearly indicate that the positive changes in personality traits can be attributed to the table tennis training intervention (Figure 2).

**Table 5:** Changes in the five dimensions of the big five personality traits (pretest to posttest).

Dimension	Groups	Pretest to posttest difference (Mpost–Mp)	*T*	*P*	Cohen’s *d*
Neuroticism	Experimental group	−0.35	−4.56	<0.001	−0.89
	Control group	−0.04	−0.87	0.387	−0.10
Agreeableness	Experimental group	+1.29	15.67	<0.001	1.56
	Control group	+0.12	1.45	0.151	0.15
Openness	Experimental group	+1.92	12.34	<0.001	1.67
	Control group	−0.19	−1.23	0.223	−0.12
Extraversion	Experimental group	+0.86	8.45	<0.001	0.86
	Control group	−0.56	−3.21	0.02	−0.46

## 4 Discussion

The impact of table tennis practice on the Big Five personality traits of primary school students was the central focus of this study. Comparative analysis revealed no significant changes in any of the Big Five dimensions within the control group between pretest and posttest measurements. This finding indirectly suggests that routine school physical education may not fully realize its potential in cultivating positive character and fostering holistic personality development in students. In contrast, following systematic table tennis training, the experimental group demonstrated significant improvements across all Big Five dimensions except for Conscientiousness. This indicates that table tennis may, to some extent, positively influence students’ emotional functioning—an outcome of critical importance for primary school students during their formative personality development stage. However, the intervention did not appear to substantially enhance students’ self-control abilities, as reflected in the Conscientiousness dimension. Analysis of the results revealed that, compared to the control group, scores for the experimental group changed across the personality dimensions, with specific patterns outlined as follows.

### 4.1 Emotional stability has significantly improved

The present study found that the experimental group showed a significant decrease in Neuroticism scores, with a substantial interaction effect size, indicating that table tennis training has a clear and relatively strong promotive effect on enhancing emotional stability in primary school students. After a period of systematic table tennis training, it was observed that table tennis practice can effectively regulate students’ mental stress, alleviate anxiety, and positively influence the mitigation of negative emotions. This aligns with findings in the field of sport psychology, where research suggests that structured programs with built-in pressure constraints help adolescent athletes develop better psychological skills (Tassi et al., 2023).

Furthermore, a systematic review indicated that participating in team sports three or more times per week can reduce the incidence of depressive symptoms in adolescents by 30% (Biddle and Asare, 2011). In addition, adolescents engaging in physical activity three or more times per week demonstrate significant improvements in Neuroticism (Lacinova et al., 2023). The specialized table tennis training implemented in this study supports this perspective. As an open-skill sport, table tennis, with its rapid rally-based competition, immediate win-loss feedback, and continuous cognitive-motor coordination demands, provides participants with intensive “micro-scenarios” for coping with pressure and setbacks. Within these scenarios, children learn to manage competitive anxiety, regulate emotional fluctuations after mistakes, and experience the sense of achievement that comes from regaining control of the game. This may represent an effective pathway for the development of emotion regulation abilities (González-Devesa et al., 2024). Notably, team sports, which share similarities with table tennis in terms of social dynamics, may have superior effects on improving neuroticism compared to individual sports, as they offer richer social interaction contexts (Holt et al., 2017).

### 4.2 Enhancing open-mindedness and willingness to explore

The significant increase in Openness scores observed in the experimental group suggests that table tennis practice can stimulate children’s curiosity, imagination, and receptivity to new experiences. Analysis of intergroup comparisons indicated that the students in the experimental group demonstrated broadened thinking, enhanced imagination, and greater curiosity toward novel stimuli. This implies that school-based table tennis training can effectively enhance cognitive engagement in primary school students, potentially fostering positive changes in their daily lives and academic learning. Existing research indicates that table tennis demands continuous anticipation of opponents’ intentions, creative shot placement, and real-time tactical adjustments. This dynamic sporting environment enhances pattern recognition, thereby promoting the development of creative problem-solving strategies (Memmert, 2015). Since creativity is a sub-dimension of open-minded thinking, this evidence supports the notion that the dynamic context of table tennis positively influences the Openness dimension. Furthermore, both openness and innovativeness rely on robust executive functions (e.g., mental simulation and the inhibition of rigid thinking). A study on children’s cognition and activity confirmed that open-skill training, which requires rapid decision-making and adaptive responses, can effectively stimulate plasticity in the cognitive system (Alvarez-Bueno et al., 2017). Previous research has also explicitly stated that physical exercise can promote divergent thinking in athletes (Colzato et al., 2013), aligning with the intervention in this study, where table tennis practice was designed to enhance openness by improving executive function and divergent thinking. Within the context of physical activity, choosing a preferred mode of exercise may lead to better performance and greater degrees of improvement (Ryan and Deci, 2000; Teixeira et al., 2012).

### 4.3 Development of interpersonal harmony and cooperative tendencies

Despite often being categorized as an individual sport, the significant improvement in Agreeableness scores observed in the experimental group highlights the important social dimensions inherent in table tennis practice. The training environment, which includes doubles cooperation, ritualized interactions with opponents and coaches before and after matches, and the atmosphere of team competition, collectively fosters a micro-social environment that emphasizes respect, trust, and collaboration (Marchese et al., 2022; Eime et al., 2013). Furthermore, prior research has demonstrated that team-based cooperative sports can cultivate patience and tolerance while reducing social isolation and boosting confidence (Darongkamas et al., 2011). Within these structured social interactions, children learn to abide by rules, show consideration for peers, and navigate the dynamics of competition and cooperation, thereby fostering the development of agreeableness traits. This finding supports the concept of “sport as a social skills gymnasium,” suggesting that specific sporting contexts can provide unique settings for developing prosocial attitudes and behaviors (Fraser-Thomas et al., 2005).

### 4.4 Promoting the effect of social proactivity

The experimental group demonstrated a positive improving trend in the Extraversion dimension, with a significant interaction effect. This indicates that table tennis training can, to a certain extent, enhance children’s sociability and social initiative, likely stemming from necessary communication during training and competition, peer encouragement, and a sense of collective belonging. However, compared to typical team ball sports, table tennis involves relatively limited sustained high-intensity verbal interaction and complex role-based interplay; its social patterns are more intermittent and task-oriented (Allen et al., 2013). Nevertheless, its role in promoting social engagement cannot be overlooked. Research has shown that physical activity plays a key role in reducing shyness and facilitating social skill learning, and participation in sports can promote the development of social skills in children and adolescents (Shameli et al., 2013; Haugen et al., 2013; Erdilanita and Ma’mun, 2025). In another study aimed at improving the socio-emotional competence of children with autism, which also employed sports as an intervention method, the results showed that participants’ socio-emotional skills were effectively enhanced after the intervention. This further indicates that sports intervention can promote communication abilities and social skills (Levante et al., 2024). Consistent with the findings of the present study, after a period of table tennis practice, students in the experimental group exhibited good communication and interaction skills within the sport, confirming that table tennis practice effectively promotes students’ extraversion.

### 4.5 Rigorousness faces challenges in its formation

A noteworthy finding was the absence of a significant “Group × Time” interaction effect for the Conscientiousness dimension. This indicates that the 16-week table tennis training, which focused on skill acquisition and competitive performance, did not produce a uniquely superior impact compared to the control condition on primary school students’ orderliness, sense of responsibility, or self-discipline. A possible explanation is that, although sports training inherently involves rule adherence and repetitive practice, its transfer effect on general conscientious traits may be limited if the program design does not explicitly incorporate cognitive-behavioral strategies targeting self-regulation, such as goal setting, self-monitoring, and plan execution (Bogg, 2008; Beck et al., 2011). Future sports intervention studies aiming to improve conscientiousness could consider more explicitly integrating mental skills training modules (e.g., goal management and habit formation strategies) into physical education curricula.

### 4.6 Differences observed in the control group

Furthermore, the control group exhibited declines in Agreeableness and Openness levels. The study hypothesizes that this may be because students in the control group lacked regular habits of sports participation. Consequently, during the experimental period, they may have experienced sports-related pressure, which consumed their psychological energy and self-control resources. This phenomenon aligns with the “ego depletion” principle, which posits that an individual’s active volition is a limited internal resource (Baumeister et al., 2018). From a goal-directed perspective, students unaccustomed to regular sports participation are less likely to adopt “sports benefits” as a goal orientation. This can lead individuals to consciously or unconsciously suppress behaviors that differ from their core objectives. Existing research indicates that under such “goal shielding,” individuals tend to inhibit attention to other competing goals (Shah et al., 2002). Regarding the observed decline in Agreeableness, children in this age group are experiencing an expansion of their social circles, which is often accompanied by increased peer competition and conflict. In the absence of structured, guided group activities (such as sports teams), they may be more prone to forming cliques, exhibiting exclusionary behaviors, or struggling to balance competition and cooperation (Rubin et al., 2006). The control group lacked the “social skills gymnasium”—namely, systematic physical activity—available to the experimental group (Fraser-Thomas et al., 2005; Vella et al., 2014). As for the decrease in Openness, this may be attributed to the gradually increasing academic pressure experienced in schools during this period. Teaching methods may place greater emphasis on standardized answers and knowledge transmission rather than fostering curiosity, imagination, and exploratory thinking. Such an environment could potentially inhibit the development of openness (Gopnik, 2012; Beghetto and Kaufman, 2014). If the leisure time of children in the control group is not directed toward activities such as sports or the arts, it may be more occupied by passive entertainment (e.g., watching television and playing simple video games). These activities generally require minimal active thinking or creative engagement (Twenge and Campbell, 2018; Lillard and Peterson, 2011).

### 4.7 Research limitations and future directions

This study provides preliminary causal evidence that table tennis practice promotes the development of key personality traits—particularly emotional stability, openness, and agreeableness—in primary school students. It supports the value of incorporating such structured sports as an important vehicle for promoting children’s mental health and personality education. However, several limitations should be acknowledged. First, the quasi-experimental design, despite the use of propensity score matching, cannot fully rule out the influence of unmeasured confounding factors. Second, the sample consisted exclusively of male primary school students, limiting the generalizability of the findings to female populations and other age groups. Third, the reliance on self-report measures suggests that future studies could benefit from incorporating multi-source data, such as teacher evaluations and behavioral observations. Finally, the study lacked a fine-grained analysis of specific components within the training content, such as the proportion of different training elements.

Future research could verify these findings through large-scale randomized controlled trials and conduct long-term follow-up to examine the sustainability of the effects. In addition, studies could delve deeper into the mechanisms linking different elements of table tennis training—such as cognitive load, types of social interaction, and competitive intensity—to changes in specific personality dimensions. This would provide more precise scientific evidence for designing targeted intervention programs that integrate sports and education.

## 5 Conclusion

This study systematically elucidates the shaping effects of table tennis practice on the Big Five personality traits of primary school students. The results demonstrate that table tennis, as a highly interactive and dynamic sport, promotes personality development through multiple pathways. Firstly, mechanisms of teamwork and role rotation—such as tactical cooperation and on-court positional responsibilities—provide students with rich scenarios for social practice, significantly enhancing extraversion. This manifests as increased proactive communication, improved leadership, and reduced social anxiety, which aligns closely with Holt et al.’s (2017) “sport-based social learning model,” whereby dynamic team environments strengthen social cognitive abilities through role-taking (Lacinova et al., 2023). Secondly, stress adaptation and emotion regulation constitute a core pathway for improving neuroticism (Jeronimus et al., 2016; Widiger and Oltmanns, 2017). The win-loss situations and immediate feedback mechanisms inherent in table tennis matches gradually enable students to acquire emotional management strategies, such as utilizing team support to mitigate frustration, supporting Agbaria and Mokh (2022) interaction theory on “extraversion buffering high neuroticism”. Thirdly, open-ended problem-solving and creative thinking are activated during tactical training (Memmert and Roth, 2007). Tasks such as improvisational breakthroughs and spatial anticipation significantly elevate the Openness dimension, consistent with Memmert’s (2015) emphasis on “dynamic environments fostering tactical creativity”. However, the study also found limited promotion of the Conscientiousness dimension (e.g., self-discipline and goal persistence) through table tennis practice, possibly because adolescents in competitive contexts tend to focus more on immediate incentives rather than long-term planning.

From an educational practice perspective, school-based table tennis training serves not only as a vehicle for physical conditioning but also as a significant medium for personality education. This study confirms that systematic table tennis practice, through multidimensional social interaction and cognitive challenges, significantly promotes the development of extraversion, openness, agreeableness, and emotional stability in primary school students, providing empirical support for the “sport-education integration” strategy. Future research could employ interdisciplinary methods—such as integrating sport psychology and neuroscience—to further uncover the deeper mechanisms through which sports interventions influence personality plasticity.

## Data Availability

The raw data supporting the conclusions of this article will be made available by the authors, without undue reservation.
